# Dislocation of the Elbow, of Five Months and Thirteen Days Standing

**Published:** 1847-02

**Authors:** Daniel Brainard

**Affiliations:** Prof. of Surgery in Rush Medical College, Chicago


ARTICLE V.

*Dislocation of the Elbow, of Five Months and Thirteen Days standing. Reduction.* By DANIEL BRAINARD, M.D., Prof. of Surgery in Rush Medical College.

On the 14 Dec. 1846, Mr. G. æt. 19 years, consulted me about his left elbow, which had been injured on the 2d July last. A surgeon was called at the time, who pronounced the injury a dislocation, and, as he supposed, reduced it. After carrying the member in a sling in a bent position for about three weeks, the Dr. directed him to "move it and straighten it." Such was the account he gave.

On examination, the following appearances were observed. The inferior extremity of the os humeri was found resting upon the anterior surface of the bones of the fore-arm at the elbow, the condyles and articular surfaces being distinctly felt. The radius and ulna were as distinctly felt, resting behind upon it, the fore-arm being extended, shortened about an inch, and incapable of being flexed in the slightest degree by moderate force. The muscles of the member were much atrophied for want of use, and the position of the bones could readily be recognized by the eye, as well as by the touch, so that no one could at that time fail of detecting a dislocation of both bones of the fore-arm backwards.

If it was easy to detect the nature of the accident, it was more difficult to determine upon the propriety of attempts at reduction. For ancient dislocations of the elbow have not received the same attention from surgeons as those of the shoulder; and no rules have been generally adopted in regard to the length of time at which efforts for reduction may be made with prospect of success.

In my own experience I have only met with one case of the kind, suitable for reduction. This was of eight weeks' standing, in a girl about six years of age, and, in reducing it, the triceps muscle was found to resist so firmly that it was divided by subcutaneous section; after which the reduction was easily effected, and the result was favorable. The force required was, however, so great as not to make me think favorably of a similar attempt at a much later period. The importance of the object to the patient, however, inclined me to make the effort, which was accordingly done on the 15th inst., with the assistance of Professors McLean, Blaney and Dr. Maxwell, in presence of the medical class and several physicians.

Seating the patient upon a chair, I flexed the fore-arm around my knee, drawing it forward, at the same time, until it was brought to about its natural limit of flexion. This required considerable force. On examination, it was found that the bones were brought down to the natural position, except that the articular surface of the os humeri did not seem to be fully received into the greater sigmoid cavity of the ulna. The effusion of blood which took place at the time of the accident, and the subsequent inflammation had undoubtedly left behind, upon these articular surfaces, quantities of lymph which prevented their nearer contact. Desirous of remedying this as much as possible, force was moderately applied with the pullies to press the ulna forwards, while the humerus was drawn backward.

This produced a decided improvement, without entirely effecting the object in view. A roller was applied about the hand and fore-arm, with compresses upon the elbow in such a manner as to prevent the danger of a reproduction of the displacement, the fore-arm placed in a sling in a bent position, and evaporating lotions applied. Considerable swelling occurred, which, at the end of a week, had subsided sufficiently to allow of passive motion for restoring the movements of the joint. Flexion and extension are now made to a great extent, and the bones have come very nearly to their natural position; so that no doubt exists that with good care and perseverance the use of the member will be regained, and with little if any defect in the joint.

So favorable a result with so little force was far from being anticipated. But it should be added, that the patient was not very muscular; and that the system was in a state of debility and relaxation, in consequence of intermittent fever, under which he had labored during the autumn. The care which had been taken to keep the joint extended also evidently favored reduction, since it was only necessary to use the fore-arm as a lever in flexion to bring the bones to their proper places.

Without these favoring circumstances, it is obvious that much greater difficulty might have been experienced in the reduction.

*Chicago, Dec.* 31, 1846.

